# Blood pressure lowering to prevent dementia, the role or frailty, when not to treat?

**DOI:** 10.1002/alz70860_098290

**Published:** 2025-12-23

**Authors:** Xiaoying Chen, Katie Harris, Wang Ruirui, Linan Chen, Craig S. Anderson, Nigel S. Beckett, Christopher J. Bulpitt, John Chalmers, Françoise Forette, Kenneth Rockwood, Jan Staessen, Christophe Tzourio, Jeff Williamson, Mark Woodward, Ruth Peters

**Affiliations:** ^1^ The George Institute for Global Health, Sydney, NSW, Australia; ^2^ George Institute for Global Health, UNSW, Sydney, NSW, Australia; ^3^ University of New South Wales, Sydney, NSW, Australia; ^4^ Guys and St Thomas' NHS Trust, London, United Kingdom; ^5^ Imperial College London, London, United Kingdom; ^6^ International Longevity Centre, Paris, France; ^7^ Dalhousie University, Halifax, NS, Canada; ^8^ Alliance for the Promotion of Preventive Medicine (APPREMED), Mechelen, Belgium; ^9^ Bordeaux Population Health Research Center, Inserm U1219, University of Bordeaux, Bordeaux, France; ^10^ Wake Forest University School of Medicine, Winston‐Salem, NC, USA; ^11^ George Institute for Global Health, Imperial College London, London, United Kingdom; ^12^ The George Institute for Global Health, University of New South Wales, Imperial College London, Sydney, NSW, Australia

## Abstract

**Background:**

High blood pressure(HBP) is a risk factor for dementia. Clinical trials show antihypertensive treatment in those ∼60‐80 years lowers risk of incident dementia.^1^ However, observational data^2^ show differing patterns of benefit/risk for HBP and dementia in older age which may relate to the confounding influence of frailty. Frailty is a syndromal diagnosis that reflects a loss of resilience, and exacerbates expression of dementia pathology.^3,4^ Since varying levels of frailty are present in clinical trial participants, we sought to examine its role as a potential moderator of the impact of antihypertensive treatment on incident dementia.

**Method:**

Single‐stage individual‐participant‐data meta‐analysis. Data were merged from four landmark double‐blind placebo‐controlled trials of antihypertensive drugs with blinded adjudicated dementia endpoints. Frailty was assessed using a robust tool, the frailty index(FI) where scores range from 0.01 to 1.00. Frailty was modelled as a continuous and binary variable FI≤0.21 for none/mild frailty and FI>0.21 moderate‐severe frailty^5^. A multilevel multinomial regression model was used to determine the impact of baseline frailty on the impact of antihypertensive treatment on incident dementia taking account of the competing risk of death, unadjusted and adjusted for age, sex, education.

**Result:**

Data on dementia and frailty were available for 24122 participants (mean age 68.5(SD9.31)yr, female 44%) with a median follow up of 4.5 years. Baseline FI median score was FI 0.16 (interquartile interval 0.11‐0.23). Adjusted analyses: There was no interaction between frailty (continuous) and antihypertensive treatment and incident dementia (*p* = 0.47). For those an F ≤.21 the odds ratio for the impact of antihypertensive treatment was of 0.88(0.72,1.07) compared to 0.85(0.65,1.10) for an FI>0.21. Unadjusted results were similar.

**Conclusion:**

Antihypertensive treatment is likely to reduce incident dementia in those aged∼60‐80 across varying levels of frailty. However, since clinical trial populations are biassed towards less frail participants, there are limitations to the generalizability of these data to more severe frailty in the general population. Further analyses will allow the investigation of the frailty/antihypertensive/dementia relationship in more detail. These data have implications for treatment guidelines for cardiovascular prevention alongside approaches to the management of people at high‐risk of dementia.

1. Eur Heart J 2022;43(48):4980–90

2. JAMA Intern Med;2022;182(2):142‐52

3. Lancet Neurol;2018;18(2):17784

4. Nat Aging;2021;1:651–65

5. Lancet Health Longev;2023;2(2)e96‐e104

